# Perinatal Mortality in South Asia: Systematic Review of Observational Studies

**DOI:** 10.3390/ijerph15071428

**Published:** 2018-07-06

**Authors:** Pramesh Raj Ghimire, Kingsley E. Agho, Blessing J. Akombi, Nidhi Wali, Michael Dibley, Camille Raynes-Greenow, Andre M. N. Renzaho

**Affiliations:** 1 School of Science and Health, Western Sydney University, Locked Bag1797, Penrith, NSW 2571, Australia; K.Agho@westernsydney.edu.au (K.E.A.); blessingakombi@yahoo.com (B.J.A.); 2 School of Social Sciences and Psychology, Western Sydney University, Locked Bag1797, Penrith, NSW 2751, Australia; N.Wali@westernsydney.edu.au (N.W.); Andre.Renzaho@westernsydney.edu.au (A.M.N.R.); 3 Sydney School of Public Health, The University of Sydney, Edward Ford Building (A27), Sydney, NSW 2006, Australia; michael.dibley@sydney.edu.au (M.D.); camille.raynes-greenow@sydney.edu.au (C.R.-G.)

**Keywords:** perinatal mortality, South Asia, systematic review, public health

## Abstract

Background: This study aimed to systematically review observational studies on perinatal mortality in South Asia. Methods: This review was conducted according to the Preferred Reporting Items for Systematic reviews and Meta-Analyses (PRISMA) guidelines. Five computerized bibliographic databases: MEDLINE, CINAHL, Embase, PsycINFO, and Scopus were searched for published studies which reported factors associated with perinatal mortality in South Asia from 1 January 2000 to 20 March 2018. All relevant observational studies (cohort, cross-sectional and case-control) were reviewed. Results: Fourteen studies met the selection criteria. The most common factors associated with perinatal mortality were: low socioeconomic status, lack of quality health-care services, pregnancy/obstetric complications and lack of antenatal care. Conclusions: Interventions to reduce perinatal mortality in the South Asia should focus on the provision of adequate antenatal care and quality healthcare services which are accessible to women of low socioeconomic status.

## 1. Introduction

Perinatal mortality is a major public health challenge in many low- and middle-income countries (LMICs). Perinatal mortality refers to fetal death after 28 weeks of gestation and before the 7th day of life [1]. In LMICs, perinatal mortality has been reported to be associated with inadequate access to quality care services [2], inadequate infant nutrition [3], and suboptimal environmental conditions such as unsafe water supply, inadequate sanitation, and poor housing facilities [4]. Almost half of the stillbirth and early neonatal mortality occurs during the period of labor and delivery [5]; with prematurity, low birth weight, obstructed labor, pregnancy complications and infections identified as the leading causes for these untimely deaths [6,7,8].

Globally, the number of perinatal deaths decreased from 5.7 million in 2000 to 4.1 million in 2015 [9]. However, 95% of these deaths occurred in LMICs with the largest numbers reported in South Asia and sub-Saharan Africa [9,10]. Despite the substantial global progress in improving child survival [10], perinatal mortality remains an urgent public health concern and progress made has been slower than that reported for maternal and child mortality [10,11,12,13]. Thus, reducing inequities among the most vulnerable pregnant women and newborns is an important strategy in achieving the Sustainable Development Goal (SDG) target of ending preventable perinatal deaths [14,15].

Previous studies conducted in South Asia identified distal determinants such as maternal age [16], poor socio-economic status [17], illiteracy [18], obesity and overweight [19], and poor water and sanitation [20] to be significantly associated with increased perinatal mortality. The 2014 Lancet series on Every Newborn suggested that annually 33% stillbirth can be averted with increased coverage and quality interventions such as antenatal care and skilled birth attendant services, detection and management of pregnancy-induced disorders as well as intrauterine growth restriction, and management of preterm labor [21]. In addition, the World Health Organization (WHO) recommends community-based cost-effective newborn care interventions such as immediate drying and additional stimulation, dry cord care, skin to skin contact in the first hour of life, and immediate breastfeeding [22] to reduce newborn death. However, these interventions are primarily focused on minimizing the risk factors which are prevalent around the perinatal period but do not take into cognizance the distal determinants responsible for increased perinatal mortality [16,17,18,19,20,23]. Previous studies conducted on perinatal mortality in developing countries reported factors such as maternal anemia [24], institutional delivery [25] and antenatal care services [26] as significantly associated with increased mortality without taking into account the predisposing socioeconomic and environmental level factors. Hence, there is a need to understand the most significant community-, household-, environmental-, and socioeconomic-related factors associated with perinatal mortality to guide the formulation of effective policies and programs to accelerate progress for newborn survival across South Asia.

Presently, no study has collectively and systematically analyzed the predisposing factors at the individual-, community-, household-, environmental-, and socioeconomic levels associated with perinatal mortality in South Asia to drive context-specific interventions which will lead to a decline in preventable perinatal death within the region. Hence, the aim of this study was to systematically review the factors associated with perinatal mortality in South Asia, thus contributing to the body of evidence needed to inform effective policy strategies to reduce perinatal mortality, and setting the region on the path to achieving the SDG target by reducing perinatal mortality to as low as 12 deaths per 1000 births by 2030 [14,15].

## 2. Materials and Methods

### 2.1. Outcome Measure

The outcome measure for this study was perinatal mortality which refers to the number of stillbirths and deaths in the first week of life [1].

### 2.2. Search Strategy

This systematic review was conducted according to the Preferred Reporting Items for Systematic reviews and Meta-Analyses (PRISMA) guidelines [27]. Relevant MeSH headings and keywords were generated and used to extensively search five bibliographic databases: MEDLINE, CINAHL, Embase, PsycINFO, and Scopus for peer-reviewed articles published between 1 January 2000 and 20 March 2018. The year 2000 was used as a baseline for this review because this was the beginning of the Millennium Development Goals (MDGs) and hence will aid in tracking the progress of the region in line with the MDGs.

Retrieved articles from each database were imported into an EndNote library. To capture relevant publications that might have been omitted, a further search of the bibliographical references of all retrieved articles that met the inclusion criteria was performed, complemented by citation tracking using Google Scholar.

The search strategy was developed using Boolean operators for three major concepts: perinatal mortality, risk factors, and countries in South Asia. The following combination of keywords was used for the search:

[“perinatal mortality*” or “Perinatal death*”]
AND
[risk* or “risk factor*” or predictor* or determinant* or socioeconomic* or sociodemographic* or factor*]
AND
[“South Asia*” or “Southern Asia*” or Afghan* or Bangladesh* or Bhutan* or India* or Maldives or Nepal* or Pakistan* or Sri Lanka*]

### 2.3. Inclusion and Exclusion Criteria

Eligibility assessment was conducted and studies were included in this review if they (i) focused on perinatal mortality; (ii) were conducted in South Asia; (iii) reported factors associated with perinatal mortality; (iv) were published between 1 January 2000 and 20 March 2018; (v) were observational studies (cross-sectional studies, cohort studies and case-control studies); (vi) were published in a peer-reviewed journal (non-peer reviewed research, reviews, commentaries, letters to editors and conference presentations were excluded); (v) written in English. The inclusion of eight South Asian countries (Afghanistan, Bangladesh, Bhutan, India, Maldives, Nepal, Pakistan, and Sri Lanka) in our study is based on UNICEF regional classification [28].

### 2.4. Data Extraction

All articles identified from the search of each database were imported into an EndNote library and duplicates were eliminated. The first author (PRG) independently read and screened the titles and abstracts of all retrieved articles. In the final screening phase, full-texts of selected articles were identified by PRG using electronic databases, school library and contacting author via email, and studies which met the eligibility criteria were retained after reading the full text. All data extraction and appraisals of retrieved studies were independently reviewed by PRG, BJA and NW, and all disagreements among the three reviewers were resolved through discussion. The summary of the selected studies was recorded, and this included: author, year of publication, country of publication, study design, pregnancy outcome(N), factors associated with perinatal mortality, study limitations, and quality assessment score (Table 1).

### 2.5. Quality Assessment

The quality of all selected studies was assessed using the National Institute of Health (NIH) Study Quality Assessment Tools for observational studies [38]. The tools consist of criteria which evaluate the internal validity of studies by considering the potential risk of selection bias, information bias, measurement bias, and confounding. Case-control studies were assessed using 12 criteria, while cohort and cross-sectional studies were assessed based on 14 criteria. The reviewed studies were assigned a quality score on a scale of 0–12 points for case-control studies and 0–14 points for cohort and cross-sectional studies (0 if the study did not meet any criteria and 12 or 14 points if the study met all the criteria for the appropriate study design). The sum of points indicated the overall quality of a study. Studies were rated as poor quality (score ≤ 4); fair quality (5–9); and good quality (≥10) as shown in Supplementary Tables S1 and S2.

## 3. Results

**Figure 1:**
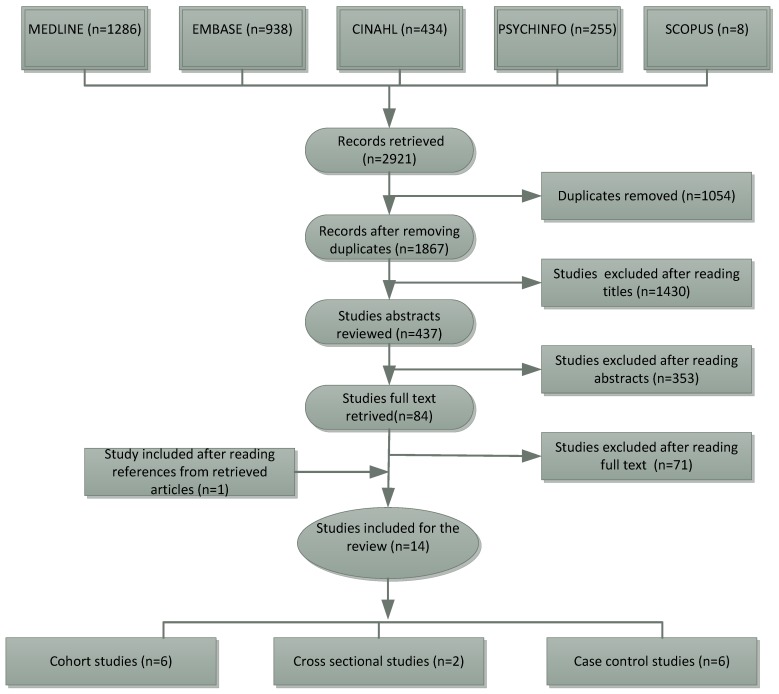
The flow chart for study selection based on PRISMA 2015 guidelines.

A total of 2921 articles were retrieved from the five databases. After applying the selection criteria at each screening stage, a total of 14 articles were retained (Figure 1).

### 3.1. Characteristics of Selected Studies

Four studies were conducted in Pakistan, 4 studies in Bangladesh, 4 studies in India, 1 study in Afghanistan and 1 study in India and Pakistan (Table 1). There were no studies from Nepal, Maldives, Sri Lanka and Bhutan. The sample size of selected studies ranged from 55 to 57,108 women or pregnancies. Of the 14 studies selected; 6 were cohort studies [23,29,31,33,35,37], 2 were case-control studies [18,32] and 6 were cross-sectional studies [16,17,19,30,34,36]. Based on the NIH criteria, 1 study was of good quality [29], while 10 studies were of fair quality [16,17,18,19,23,32,33,34,36,37], and 3 studies were of poor quality [30,31,35]. The details of specific scores assigned to each quality assessment domain are provided in Supplementary Tables S1 and S2.

### 3.2. Summary of Reviewed Studies

Low socioeconomic status was found to be associated with perinatal mortality as reported in studies conducted in India [17,18] and Bangladesh [29] (Table 1). A cross-sectional study [17] and a case-control study [18] conducted in India also reported that uneducated women were more susceptible to perinatal mortality compared to those who were educated. Furthermore, a cohort study conducted in Pakistan reported that perinatal mortality was higher among women who reside in rural areas compared to those residing in urban areas [35].

In this review, suboptimal maternal anthropometry such as low maternal body weight and height [32] as well as maternal obesity and overweight [19,37], were found to be associated with perinatal mortality in India [32], Bangladesh and Pakistan [19,37]. Maternal medical conditions, birth and pregnancy complications such as anemia [23,29], antepartum hemorrhage [31,36], hypertensive disorders [31], congenital anomalies [31], placenta and cord abnormalities [18], pregnancy-induced hypertension [36], probable infection [36], and neonatal and intrapartum complications [18,34] were also reported to be associated with perinatal mortality in studies conducted in Pakistan [23,31], Bangladesh [29,36] and India [18,34]. A cohort study conducted in Pakistan reported that older maternal age (≥40 years) was associated with perinatal mortality [33], while another cross-sectional study conducted in Bangladesh identified young maternal age (≤18 years) to be associated with perinatal mortality [16]. A case-control study conducted in India reported that parity of three and above was associated with perinatal mortality [18]. Another study conducted in India found that mothers having their first birth were more susceptible to perinatal mortality [17]. Multiple pregnancies were also reported to be associated with perinatal mortality [34]. Furthermore, a cohort study conducted in Bangladesh found that women having five or more pregnancies prior to the index pregnancy were associated with perinatal mortality [29].

Home birth [18,30,32], pregnancy interval [18,30,32] and history of previous death [18] were also reported to be associated with perinatal mortality. A cross-sectional study conducted in India found that mothers who were victims of domestic violence were more susceptible to perinatal mortality [17]. Another case-control study conducted in India found that women who consumed tobacco were more predisposed to perinatal mortality [18] than women who did not consume tobacco.

Low birth weight [18], small gestational size at birth [34] and prematurity [35] were found to be associated with perinatal mortality in studies conducted in India. A case-control study conducted in India [18] and a cohort study in Pakistan [35] also reported that poor antenatal care was associated with perinatal mortality.

## 4. Discussion

This review appraises the methodological quality of reviewed observational studies. We used observational studies because randomized controlled trials are not feasible and only data from observational studies are available for review [39]. Findings from this study revealed that the most common factors associated with perinatal mortality in South Asia were: low socioeconomic status, lack of quality health care services, pregnancy and or obstetric complications, and lack of antenatal care.

Socioeconomic status (SES) was reported to be associated with perinatal mortality in South Asia. Women with low SES have poorer nutrition and less access to quality maternal and child health care services which adversely impacts fetal and newborn health. Previous studies conducted in low [40,41] and high income countries [42] have reported that higher SES has a protective effect on perinatal mortality, and this maybe as a result of better access and utilization of quality healthcare services such as skilled birth attendants, antenatal care, postnatal care, and institutional deliveries [7,25,43]. The utilization of antenatal care and institutional birth that provide high-quality care are well established as interventions to reduce perinatal mortality [21,25,44], and are more likely to be accessed by women from higher SES. A recent Nepalese study indicated that women of the lower socioeconomic background were significantly less likely to use institutional birth [45] and quality antenatal care [46] resulting in higher perinatal mortality.

In South Asia, countries such as India, Pakistan and Bangladesh have a health care system which is primarily financed by out-of-pocket money [47]. This may pose a great barrier to the access and utilization of quality health care services by women particularly with low SES.

In a case-control study conducted in Kuwait, it was inferred that access to free maternal health care services for women of lower SES had a significant positive impact in reducing perinatal mortality [48]. Furthermore, a low rate of perinatal mortality was reported in Sri Lanka (<10 per 1000 births) [9] which may be due to the implementation of clear policies through well-structured community-based and institutional healthcare service delivery that provides free of charge quality maternal and child health care services irrespective of socioeconomic class [49].

In this study, lack of maternal health care services such as antenatal care and home birth were reported to be associated with perinatal mortality. The presence and accessibility of quality health care service greatly influence maternal and child health outcomes. A large number of avoidable perinatal deaths are due to inadequate health care services rendered from poorly equipped health care centers with inadequate diagnostic tools and suboptimal maternal care services. Perinatal care is intimately linked to maternal and newborn survival, and thus effective care throughout the continuum of pregnancy, labor, and into the postpartum period is essential [50]. A meta-analysis of estimating perinatal mortality by place of delivery conducted in Sub-Saharan Africa reported that 14 perinatal deaths per 1000 births could be averted if birth occurred at a high-quality health facility [25]. Hence, to reduce perinatal mortality, an improvement in the quality and access of health care services is critical [51].

In this study, maternal obstetric complications such as gestational diabetes, anemia, hypertensive disorders, preterm labor, and intrauterine growth restriction were found to be associated with perinatal mortality in South Asia. These conditions can be identified in the antenatal period, thus reinforcing the need to improve the continuum of care between antenatal identification and subsequent management of complications in health facilities [21,52]. Hence, high coverage quality antenatal care and institutional delivery/skilled birth attendants have become a part of global and country level strategy to improve birth outcome [15,53,54].

In South Asia, countries such as Bangladesh, India, Nepal and Pakistan have existing public health policies which include conditional cash transfer and voucher schemes aimed at promoting antenatal, delivery and postnatal care for women of lower socioeconomic status [47]. However, despite these policies, perinatal mortality is still high within the region which indicates that these policies have not been effective in improving health outcomes [47,55]. Hence, further research into the quality of antenatal and delivery care services offered to women is needed.

### 4.1. Strengths and Limitations

This review is a comprehensive search of the existing literature to report factors associated with perinatal mortality across South Asia. However, this study also had some limitations. First, qualitative studies were excluded from this review. The inclusion of qualitative studies in systematic reviews provides alternative explanations and enables triangulation of findings [56,57]. Second, relevant studies may have been published in a language other than English, and hence were missed in our study. Third, there were no studies from Bhutan, Maldives, Sri Lanka and Nepal on perinatal mortality; hence, more research on perinatal mortality should be done in these countries. Fourth, most studies retained for review were fair to poor quality which may affect the external validity of our findings. Finally, with such different data sources and limited information from some countries, this review did not report the pooled estimate for the effect of each factor on perinatal mortality across all countries in the region; this is due to the fact that the factors were measured differently in each study, thus reporting an estimate for the pool effect would misrepresent the impact of the factors on perinatal mortality. Furthermore, some countries have no reported studies on perinatal mortality; hence, further research should focus on analyzing the determinants of perinatal mortality in these countries.

### 4.2. Policy Implications

Findings from this study are useful for identifying the underlying factors associated with perinatal mortality in South Asia in order to assist in the proper allocation of health resources. These findings will also assist policy makers in planning, developing and implementing of public health interventions which provide appropriate antenatal and obstetric care services aimed at improving maternal health and reducing perinatal mortality at both the individual and community levels. This study also serves as a needs assessment indicator to countries having no representation of research on perinatal mortality to further explore the factors associated with perinatal mortality within its populace.

## 5. Conclusions

This systematic review found that pregnancy complications are the major causes of perinatal mortality in South Asia. A protective effect of perinatal mortality was found in women who used antenatal care and institutional delivery as well as those with a high SES. Socioeconomic disparity remains a significant barrier to the utilization of maternal and child health services. Hence, cost-effective health care interventions such as quality antenatal care and institutional delivery are needed and should target women of low socio-economic status. Furthermore, due to there being no evidence from some countries in South Asia, there is need to improve data collection by introducing effective health information management systems (HIMS) aimed at assisting health agencies gather data on perinatal mortality to influence current and future needs for health care services.

## Figures and Tables

**Table 1:** Summary of selected studies.

Author [Ref.] Year Country	Study Design	Pregnancy Outcome (N)	Factors Associated with Perinatal Mortality	Study Limitations	Quality Assessment Score
Ahmed et al. [17] 2006 India	Cross-sectional study	2199	Domestic violence, first birth, lack of maternal education, poor socioeconomic status.	Underreporting of violence because of the involvement of perpetrators for obtaining data, no direct question to justify if the violence occurred during pregnancy, and underreport of pregnancy and death due to a retrospective study.	7/14 (Fair)
Bari et al. [29] 2002 Bangladesh	Cohort study	965	Five or more pregnancy prior to index pregnancy, assisted delivery, poor economic status, anemia prevalence.	All study variables used in the analysis are not defined.	10/14 (Good)
Guidotti et al. [30] 2009 Afghanistan	Cross-sectional study	53,524	Mode of delivery, medical risk factors.	Data were obtained from hospital records that are not primarily designed for research purpose; and this has limited study findings for the adjustment of other important confounding factors.	4/14 (Poor)
Iqbal et al. [31] 2014 Pakistan	Cohort study	11,260	Antepartum hemorrhage, hypertensive disorders, mechanical problems, congenital anomalies, neonatal problems, maternal medical problems.	Small sample size.	2/14 (Poor)
Khan et al. [19] 2017 Bangladesh	Cross-sectional study	6584	Maternal overweight and obesity.	Pregnancy outcomes reported in this study are based on based on maternal recall in five years preceding the survey that may inaccurately capture the total number of perinatal death.	7/14 (Fair)
Perveen et al. [23] 2016 Pakistan	Cohort study	234	Sideropaenic anemia.	Small sample size, hospital-based study and it has the limitation of generalization of the finding into wider community level.	5/14 (Fair)
Sachar et al. [32] 2000 India	Case-control study	2424	Lower maternal weight and height, BMI, literacy, pregnancy interval, prematurity, home delivery.	The study is based on rural setting, and findings from this study cannot be generalized to make a programmatic response to urban women. The risk variables used in the study are poorly defined.	6/12 (Fair)
Shabbir et al. [33] 2014 Pakistan	Cohort study	2010	Multiparous, advanced maternal age.	Limitation of ascertainment bias.	7/14 (Fair)
Shah et al. [18] 2000 India	Case-control study	10,715	Antenatal care, socioeconomic status, maternal education, tobacco consumption, parity, history of abortion, history of stillbirth, history of neonatal death, history of infant death, pregnancy spacing, maternal medical problems, obstetric problems, weeks of gestation, birth weight, type of labor, rupture of membranes, type of presentation, mode of delivery, anesthesia, intrapartum medical problems, Apgar score, state of amniotic fluid, resuscitation of the newborn, placenta and cord abnormalities, congenital defects.	Data collection was not regionally homogeneous limiting to apply study findings across the country.	9/12 (Fair)
Siddalingappa et al. [34] 2013 India	Cross-sectional study	314	The intrapartum complication, intrauterine complication, small gestational size at birth, the time taken for a first cry, multiple pregnancies.	Limited sample size, limited scope for generalization.	5/14 (Fair)
Wassan et al. [35] 2009 Pakistan	Cohort study	2778	Antenatal care, birth weight, gestational age, fetal causes, types of residence, maternal risk factors.	Hospital-based study and the study lacks generalizability of findings to a wider population. The study has lacked statistical measure to examine factors associate with perinatal mortality.	4/14 (Poor)
Kusiako et al. [16] 2000 Bangladesh	Cross-sectional study	3865	Maternal age, poor obstetric history, antenatal nutritional marker, signs and symptoms of pregnancy, length of gestation, complications during labor.	As the data was collected by midwives, and lack of verbal autopsy may increase classification bias for perinatal mortality.	6/14 (Fair)
Khanam et al. [36] 2017 Bangladesh	Cross-sectional study	6285	Antepartum hemorrhage, pregnancy-induced hypertension, probable infection.	The use of cross-sectional data lacks establishment of a temporal relationship between pregnancy complication and perinatal mortality. Selective recall bias as mothers who experienced perinatal deaths were more likely to recall antepartum complications compared with those who did not experience a complication.	8/14 (Fair)
Short et al. [37] 2018 India and Pakistan	Cohort study	41,778	Obesity and overweight during pregnancy.	The findings are limited to reflect the whole of the cohort as almost 60% women who measured their weight after 12 weeks of pregnancy were excluded from the analysis.	8/14 (Fair)

## Supplementary Materials

The following are available online at http://www.mdpi.com/1660-4601/15/7/1428/s1, Table S1: Quality assessment of selected cross-sectional and cohort studies, Table S2: Quality assessment of selected case-control studies.
