# Supplementary material for: Perinatal Mortality in South Asia: Systematic Review of Observational Studies
Source: Int J Environ Res Public Health. 2018 Jul 6;15(7):1428. doi: 10.3390/ijerph15071428 (PMC6069066; doi:10.3390/ijerph15071428)
Supplement: Supplementary file 1 [file ijerph-15-01428-s001.pdf]

Supplementary table 1: Quality assessment of selected cross-sectional and cohort studies

Author [Ref.] Year country	Was the research question or objective in this paper clearly stated?	Was the study population clearly specified and defined?	Was the participation rate of eligible persons at least 50%?	Were all the subjects selected or recruited from the same or similar populations (including the same time period)? Were inclusion and exclusion criteria for being in the study prespecified and applied uniformly to all participants?	Was a sample size justification, power description, or variance and effect estimates provided?	For the analyses in this paper, were the exposure(s) of interest measured prior to the outcome(s) being measured?	Was the timeframe sufficient so that one could reasonably expect to see an association between exposure and outcome if it existed?	For exposures that can vary in amount or level, did the study examine different levels of the exposure as related to the outcome (e.g., categories of exposure, or exposure measured as continuous variable)?	Were the exposure measures (independent variables) clearly defined, valid, reliable, and implemented consistently across all study participants?	Was the exposure(s) assessed more than once over time?	Were the outcome measures (dependent variables) clearly defined, valid, reliable, and implemented consistently across all study participants?	Were the outcome assessors blinded to the exposure status of participants?	Was loss to follow-up after baseline 20% or less?	Were key potential confounding variables measured and adjusted statistically for their impact on the relationship between exposure(s) and outcome(s)?
Ahmed et al. [17] 2006 India	YES	YES	NR	YES	NR	NO	NO	NA	YES	YES	YES	NA	NA	YES
Bari et al. [29] 2002 Bangladesh	YES	YES	YES	YES	NO	YES	YES	NA	YES	YES	YES	NA	NR	YES
Guidotti et al. [30] 2009 Afghanistan	YES	YES	NR	NR	NO	NO	NO	NA	YES	NO	YES	NA	NA	NO
Iqbal et al. [31] 2014 Pakistan	YES	NO	NR	NR	NR	NO	NR	NA	NO	NO	YES	NA	NR	NO
Khan et al. [19] 2017 Bangladesh	YES	YES	YES	YES	NR	NO	NO	NA	YES	NO	YES	NA	NR	YES
Perveen et al. [23] 2016 Pakistan	YES	YES	NR	YES	YES	NO	NO	NA	NO	NO	YES	NA	NR	NO
Shabbir et al. [33] 2014 Pakistan	YES	YES	YES	YES	YES	YES	NO	NA	NO	NO	YES	NA	NR	NO
Siddalingappa et al. [34] 2013 India	YES	YES	YES	YES	NO	NO	NO	NA	NO	NO	YES	NA	NR	NO
Wassan et al. [35] 2009 Pakistan	YES	YES	NR	YES	NO	YES	NO	NA	NO	NO	NO	NA	NR	NO
Kusiako et al. [16] 2000 Bangladesh	YES	YES	NR	YES	NO	NO	NO	NA	YES	NO	YES	NA	NR	YES
Khanam et al. [36] 2017 Bangladesh	YES	YES	YES	YES	NO	NO	NO	NA	YES	YES	YES	NA	NR	YES
Short et al. [37] 2018 India and Pakistan	YES	YES	YES	YES	NO	YES	NR	NA	YES	NO	YES	NA	NR	YES

Note: YES=1, NO=0, Not Applicable (NA)=0, and Not Reported (NR)=0.

Supplementary table 2: Quality assessment of selected case-control studies

Author [Ref.] Year Country	Was the research question or objective in this paper clearly stated and	Was the study population clearly specified and defined?	Did the authors include a sample size justification?	Were controls selected or recruited from the same or similar population that gave rise to the cases (including the same timeframe)?	Were the definitions, inclusion and exclusion criteria, algorithms or processes used to identify or select cases and controls valid, reliable, and implemented consistently across all study participants?	Were the cases clearly defined and differentiated from controls?	If less than 100 percent of eligible cases and/or controls were selected for the study, were the cases and/or controls randomly selected from those eligible?	Was there use of concurrent controls?	Were the investigators able to confirm that the exposure/risk occurred prior to the development of the condition or event that defined a participant as a case?	Were the measures of exposure/risk clearly defined, valid, reliable, and implemented consistently (including the same time period) across all study participants?	Were the assessors of exposure/risk blinded to the case or control status of participants?	Were key potential confounding variables measured and adjusted statistically in the analyses? If matching was used, did the investigators account for matching during study analysis?
Sachar et al. [32] 2000 India	YES	YES	NO	YES	YES	YES	NO	NO	NO	NO	NA	YES
Shah et al. [18] 2000 India	YES	YES	NO	YES	YES	YES	YES	YES	YES	YES	NA	NO

Note: YES=1, NO=0, Not Applicable (NA)=0.
